# Synergistic Antimicrobial Effect of Antimicrobial Peptides CATH-1, CATH-3, and PMAP-36 With Erythromycin Against Bacterial Pathogens

**DOI:** 10.3389/fmicb.2022.953720

**Published:** 2022-07-15

**Authors:** Yi Lu, Hongliang Tian, Runqiu Chen, Qian Liu, Kaixiang Jia, Dong-Liang Hu, Hongwei Chen, Chao Ye, Lianci Peng, Rendong Fang

**Affiliations:** ^1^Joint International Research Laboratory of Animal Health and Animal Food Safety, College of Veterinary Medicine, Southwest University, Chongqing, China; ^2^Department of Zoonoses, Kitasato University School of Veterinary Medicine, Towada, Aomori, Japan; ^3^Chongqing Key Laboratory of Herbivore Science, Chongqing, China

**Keywords:** antimicrobial peptides, traditional antibiotics, synergistic antibacterial activity, antibacterial mechanism, antibiotic resistance

## Abstract

With the increasing bacterial resistance to traditional antibiotics, there is an urgent need for the development of alternative drugs or adjuvants of antibiotics to enhance antibacterial efficiency. The combination of antimicrobial peptides (AMPs) and traditional antibiotics is a potential alternative to enhance antibacterial efficiency. In this study, we investigated the synergistic bactericidal effect of AMPs, including chicken (CATH-1,−2,−3, and -B1), mice (CRAMP), and porcine (PMAP-36 and PR-39) in combination with conventional antibiotics containing ampicillin, tetracycline, gentamicin, and erythromycin against *Staphylococcus aureus, Salmonella enteritidis*, and *Escherichia coli*. The results showed that the minimum bactericidal concentration (MBC) of CATH-1,−3 and PMAP-36 was lower than 10 μM, indicating that these three AMPs had good bacterial activity against *S. aureus, S. enteritidis*, and *E. coli*. Then, the synergistic antibacterial activity of AMPs and antibiotics combination was determined by the fractional bactericidal concentration index (FBCI). The results showed that the FBCI of AMPs (CATH-1,−3 and PMAP-36) and erythromycin was lower than 0.5 against bacterial pathogens, demonstrating that they had a synergistic bactericidal effect. Furthermore, the time-killing kinetics of AMPs (CATH-1,−3 and PMAP-36) in combination with erythromycin showed that they had a continuous killing effect on bacteria within 3 h. Notably, the combination showed lower hemolytic activity and cytotoxicity to mammal cells compared to erythromycin and peptide alone treatment. In addition, the antibacterial mechanism of CATH-1 and erythromycin combination against *E. coli* was studied. The results of the scanning electron microscope showed that CATH-1 enhanced the antibacterial activity of erythromycin by increasing the permeability of bacterial cell membrane. Moreover, the results of bacterial migration movement showed that the combination of CATH-1 and erythromycin significantly inhibits the migration of *E. coli*. Finally, drug resistance analysis was performed and the results showed that CATH-1 delayed the emergence of *E. coli* resistance to erythromycin. In conclusion, the combination of CATH-1 and erythromycin has synergistic antibacterial activity and reduces the emergence of bacterial drug resistance. Our study provides valuable information to develop AMPs as potential substitutes or adjuvants for traditional antibiotics.

## Introduction

The discovery and wide application of antibiotics is a great innovation in medicine, but the abuse of antibiotics in livestock and humans accelerates the resistance of microorganisms to antibiotics (Lewies et al., 2017). The prevalence of antibiotic-resistant microorganisms poses a great threat to farm animal and human health. It has been speculated that approximately 10 million people will die from antibiotic-resistant bacterial infection around the world per year by 2050 (Antimicrobial Resistance Collaborators, 2019). Several common zoonotic bacteria, including *Staphylococcus aureus, Salmonella enteritidis*, and *Escherichia coli*, have shown widespread antibiotic resistance, especially the emergence of the methicillin-resistant *S. aureus* (MRSA) and extended-spectrum β-lactamase (ESBL) *E. coli*. MRSA is one of the main causes of hospital and community-acquired infections, resulting in serious diseases, such as pneumonia, osteomyelitis, sepsis, endocarditis, and bacteremia (Medina and Pieper, 2016). *Salmonella* is a foodborne pathogen causing diarrhea, and it has been reported that approximately 2.8 billion *Salmonella* infection cases occur in the world every year (Gut et al., 2018). *E. coli* is an extremely common and complex zoonotic pathogen and causes bloodstream infections, urinary tract infections, and respiratory infections, resulting in severe colibacillosis (Vila et al., 2016). Beside bacteria-induced diseases, drug resistance of these bacteria seems to pose a great danger to human and animal health. Therefore, it is an urgent need to search for effective alternatives or adjuvants for antibiotics, thereby reducing the emergence of drug resistance.

Cationic host defense peptides known as antimicrobial peptides (AMPs) are key components of the innate immune system with immunomodulatory and antimicrobial activities (Peng et al., 2022). AMPs are widely expressed in different species, such as microorganisms, plants, insects, and mammals, which show broad-spectrum antimicrobial activity against bacteria, fungi, viruses, and parasites (Jenssen et al., 2006; Choi et al., 2012). They have a positively charged structure that enables interaction with negatively charged molecules on the bacterial surface, such as lipopolysaccharides, teichoic acids, and bacterial membranes. Unlike antibiotics that interaction involves in specific target, AMPs have been speculated to be unlikely to develop microbial resistance (Mookherjee et al., 2020). It has been reported that rabbit AMP (CAP18) and two bovine AMPs (BMAP-27 and BMAP-28) have good antibacterial activity against MRSA (Blodkamp et al., 2016). Chicken cathelicidins CATH-1,−2, and−3 have antimicrobial activities against multidrug-resistant bacteria, including MRSA, ESBL *E. coli*, and vancomycin-resistant *enterococci* (Veldhuizen et al., 2013). Human cathelicidin LL-37 and cecropin (1–7)-melittin A (2–9) amide (CAMA) showed good antibacterial activity against multidrug-resistant *Pseudomonas aeruginosa* without inducing strong resistance (Geitani et al., 2019). Therefore, AMPs are potential candidates as adjuvants for antibiotics to treat antibiotic-resistant bacterial infections.

Previous studies have shown that AMPs and traditional antibiotics have a synergistic antibacterial effect. For example, LL-37-derived short AMP KR-12-a5 and its analogs showed synergistic effects with conventional antibiotics against multidrug-resistant *P. aeruginosa* (Kim et al., 2017). Furthermore, the synergistic action of crab AMPs Sphistin with azithromycin and rifampicin and the combination of cecropin A2 and tetracycline enhanced the bactericidal activity against *P. aeruginosa* (Zhang et al., 2019; Liu et al., 2020). This synergistic effect of AMPs and antibiotics is considered one of the most important strategies to successfully combat different drug-resistant pathogens. Importantly, the combined use of AMPs and traditional antibiotics is very likely to reduce the amount of antibiotics and AMPs with the reduction of toxicity and side effects to the host, which results in decreased drug-resistant bacteria to improve the effectiveness of anti-infectives.

In this study, we investigated the synergistic antibacterial effects of AMPs, including chicken (CATH-1,−2,−3, and -B1), mice (CRAMP), and porcine (PMAP-36 and PR-39) in combination with conventional antibiotics containing ampicillin tetracycline, gentamicin, and erythromycin against *S. aureus, S. enteritidis*, and *E. coli*. In addition, the antibacterial mechanism of chicken CATH-1 combination with erythromycin was investigated through different assays, including time-killing curve, scanning electron microscopy (SEM), bacterial motility, and resistant induction. Our study reveals that the CATH-1 and erythromycin combination has good synergistic antibacterial activity without resistant induction, which provides a basis for the development of AMPs as adjuvant of antibiotics to treat a microbial infection.

## Materials and Methods

### Peptides and Antibiotics

All peptides used in this study were synthesized by China Peptides (Shanghai, China) using Fmoc-chemistry and purified by reverse-phase high-performance liquid chromatography to a purity >95% (Table 1). Four conventional antibiotics, including ampicillin, tetracycline, gentamicin, and erythromycin, were used and bought from Shanghai Macklin Biochemical Co., Ltd. (Shanghai, China). A stock solution of concentrations was prepared in sterile water.

**Table 1:** Characteristics of the peptides used in this study.

**Peptide**	**Amino acid sequence**	**Length**	**Charge**
CATH-1	RVKRVWPLVIRTVIAGYNLYRAIKKK	26	+8
CATH-2	RFGRFLRKIRRFRPKVTITIQGSARF	26	+9
CATH-3	RVKRFWPLVPVAINTVAAGINLYKAIRRK	29	+7
CATH-B1	PIRNWWIRIWEWLNGIRKRLRQRSPFYVRGHLNVTSTPQP	40	+7
CRAMP	GLLRKGGEKIGEKLKKIGQKIKNFFQKLVPQPEQ	34	+6
PMAP-36	GRFRRLRKKTRKRLKKIGKVLKWIPPIVGSIPLGCG	36	+13
PR-39	RRRPRPPYLPRPRPPPFFPPRLPPRIPPGFPPRFPPRFP	39	+10

### Bacterial Strains

*S. aureus* and *S. enteritidis* isolates were obtained from laboratory clinical isolates, and avian pathogenic *E. coli* was kindly provided by Professor Qingke Kong (College of Veterinary Medicine, Southwest University). Mueller–Hinton broth (MHB; Beijing Land Bridge Technology Co., Ltd., China) was used as bacterial growth media for antimicrobial activity assays, and Luria–Bertani (LB; Qingdao Hope Bio-Technology Co., Ltd., China) agar plates were used for the colony-counting assay. Bacteria were cultured at 37°C for 18 h in MHB or LB agar plates.

### Cells

Murine erythrocytes and peritoneal macrophages were obtained from wild-type (WT) C57BL/6 mice (Chongqing Academy of Chinese Material Medical, China). Briefly, erythrocytes were obtained by collecting blood from the tail vein. Peritoneal macrophages were collected by intraperitoneal injection with 2 ml of 4% thioacetate (Aiken, Tokyo, Japan). After 3–4 days, mouse peritoneal exudate cells were collected by intraperitoneal lavage and suspended in RPMI1640 + 10% fetal bovine serum (FBS). Then, cells were seeded at a density of 2 × 10^5^ cells/well in 48-well plates and maintained at a humidified 37°C incubator with 5% CO_2_. After 2 h incubation, the nonadherent cells were removed and the adherent cells were used for assays described below. This study was approved by the Institutional Animal Care and Use Committee (IACUC) of Southwest University, Chongqing, China (IACUC-20200930-05).

PK-15 cells were maintained at 37°C with 5% CO_2_ and cultured in Dulbecco's modified Eagle's medium (Gibco, CA, USA) supplemented with 10% FBS and antibiotics (100 U penicillin/ml and 100 μg streptomycin/ml). Cells were seeded at a density of 1 × 10^5^ cells/well in 48-well plates and cultured overnight before being used for assays described below.

### Antimicrobial Activity Assay

Antimicrobial activity of AMPs and antibiotics was performed by the broth microdilution assay as reported previously (Wiegand et al., 2008). Briefly, bacteria were maintained in MHB medium at 37°C and grown to mid-logarithmic growth phase before being tested. Then, 50 μl of peptides (0–80 μM) and antibiotics (0–40,960 μM) with triplicate were mixed with an equal volume of bacterial suspension (2 × 10^6^ CFU/ml) in 96-well plates and incubated for 18 h at 37°C. After incubation, minimum inhibitory concentration (MIC) was determined by the observation of turbidity, and minimum bactericidal concentration (MBC) was determined by colony counting. Then, 50 μl mixed medium was removed from wells in which visible growth was not observed and plated out on LB agar plates. After overnight culture at 37°C, survival bacteria were counted.

### Synergistic Antibacterial Activity of AMPs and Antibiotics

The checkerboard titration assay was used to determine the synergistic antibacterial effect of AMPs (CATH-1,−3 and PMAP-36) and antibiotics (ampicillin, tetracycline, gentamicin, and erythromycin) as reported previously. In brief, 25 μl 2-fold serial concentrations of AMPs (0–40 μM) were added in vertical wells, and then 25 μl 2-fold serial concentrations of antibiotics (0–40,960 μM) were added in horizontal wells in 96-well plates. Subsequently, 50 μl of bacterial suspensions (2 × 10^6^ CFU/ml) was added and incubated at 37°C for 18 h. After incubation, the plates were visually inspected for turbidity to determine the growth. Then, 50 μl mixed medium was removed from wells in which visible growth was not observed and then plated out on LB agar plates. The synergistic antibacterial activity was determined by the fractional bactericidal concentration index (FBCI). FBCI was calculated according to the formulae FBCI = (MBC of AMPs in combination/MBC of AMPs alone) + (MBC of antibiotics in combination/MBC of antibiotics alone). The FBCI values were defined as synergy for FBCI < 0.5, additivity for 0.5 < FBCI ≤ 1, indifference for 1 < FBCI ≤ 2, and antagonism for FBCI > 2.

### Time-Killing Curve Assay

The bacterial suspension was prepared as described above. Also, 50 μl AMPs (CATH-1,−3 and PMAP-36) and erythromycin dilutions at the lowest concentrations that showed synergistic effects were prepared in 96-well plates. Then, the same volume of bacterial suspension (2 × 10^6^ CFU/ml) was added and incubated at 37°C for 0, 5, 10, 20, 30, 60, 120, and 180 min. After incubation at the indicated time, 50 μl mixed medium was removed and diluted 10-fold serially in MHB and then plated out on LB agar plates. After overnight culture, colony counting was performed.

### Hemolytic Activity and Cytotoxicity Assay

The hemolytic activities of AMPs and antibiotics were determined as previously described (Song et al., 2020). In brief, whole mice blood was centrifuged at 1,500 rpm for 5 min at room temperature and then washed three times with phosphate-buffered saline (PBS) to obtain red blood cells (RBCs). Subsequently, 2% RBCs in PBS were prepared. Then, aliquots of 100 μl RBCs were mixed with 100 μl tested compounds in PBS in polypropylene 96-well microtiter plates and incubated for 1 h at 37°C. After incubation, the plate was centrifuged for 5 min at 1,500 rpm and 100 μl supernatant was transferred to a new 96-well plate to determine the absorbance at 570 nm. RBCs in PBS served as negative control while RBCs treated with 1% Triton X-100 in PBS served as a positive control. The percent of hemolysis (%) was calculated using the following formula


$$\begin{array}{l} Hemolysise\;(\%)\\ =\frac{\left({OD}_{570nm}\;of\;treated\;sample-OD_{570nm}\;of\;negative\;control\right)}{\left({OD}_{570nm}\;of\;positive\;control-OD_{570nm}\;of\;negative\;control\right)}\\ \times 100\% \end{array}$$

For cytotoxicity assay, mice peritoneal macrophages and PK-15 cells were prepared in a 48-well plate as described above and incubated with or without tested compounds for 2 h at 37°C with CO_2._ After 2 h incubation, cells were washed with a cell culture medium twice and continued to culture for 22 or 46 h. After 24 and 48 h incubation, 150 μl of 10% WST-1 reagent was added according to the protocols of the manufacturer. After 20 min incubation, absorbance was measured at 450 nm with a microplate reader (Bio-Rad, Japan) and was corrected for absorbance at 630 nm. Untreated cells were set as a control. The percent of cell viability (%) was calculated using the following formula:


$$\begin{array}{l} Cell\;viability\;\left(\%\right)\;=\frac{OD_{450nm}\;of\;treated\;sample}{OD_{450nm}\;of\;control}\times 100\% \end{array}$$

### SEM Analysis

Mid-logarithmic phase *E. coli* cells (1 × 10^8^ CFU/ml) were treated with and without CATH-1 (1/16 MBC), erythromycin (1/16 MBC), and CATH-1 + erythromycin (1/16 MBC CATH-1 + 1/16 MBC erythromycin) in 3 ml culture medium at 37°C for 6 h. After incubation, bacterial suspension was centrifuged and washed with PBS three times. Then, bacterial pellets were fixed with 1.5 ml of 2.5% glutaraldehyde in PBS at 4°C overnight. Finally, samples were sent to the Lilai biomedicine experiment center (Sichuan, China) for SEM analysis.

### Motility Assay

CATH-1 (1/4 MIC), erythromycin (1/4 MIC), and CATH-1 + erythromycin (1/4 MIC CATH-1 + 1/4 MIC erythromycin) were added to LB semisolid agar plates, and then 5 μl *E. coli* cells (1 × 10^6^ CFU/ml) aliquot were inoculated at 37°C for 18 h. After incubation, bacterial migration distance (mm) of the circular motility was measured.

### Resistance Induction *In vitro*

Drug resistance induction of *E. coli* was performed as previously reported. The *E. coli* strain was grown independently with CATH-1 (1/4 MIC), erythromycin (1/4 MIC), and CATH-1 (1/8 MIC) in combination with erythromycin (1/16 MIC) at 37°C for 30 consecutive generations. To explore whether CATH-1 + erythromycin inhibit the development of resistance in *E. coli*, a fixed concentration of CATH-1 (equivalent to 1/4 MIC) was added to the 2-fold increasing concentration of erythromycin. Bacteria from the highest concentration of drug combination were regrown, and MIC of erythromycin was measured, and then bacteria were treated again with the drug combination. The change in MIC was described by normalizing the MIC of the *n* generation to the MIC of first generation.

### Statistical Analysis

Data are represented as mean ± SEM of three independent experiments for each group (*n* = 3). One-way ANOVA was used to analyze statistical significance among different groups. Statistical significance is shown as ^*^p < 0.05, ^**^ *p* < 0.01.

## Results

### MIC and MBC of AMPs and Antibiotics

In this study, we firstly explored the MIC and MBC of AMPs (CATH-1,−2,−3, and -B1, CRAMP, PMAP-36, and PR-39) and conventional antibiotics (ampicillin, tetracycline, gentamicin, and erythromycin) against *S. aureus, S. enteritidis*, and *E. coli*. As shown in Table 2, except for CATH-B1's MICs and MBCs more than 40 μM, MICs of other AMPs against three kinds of bacteria were basically between 2.5 and 20 μM, and MBCs were similar to MICs. Gentamicin showed good antibacterial activity with low MBCs between 2.5 and 40 μM. MICs of tetracycline were between 5 and 40 μM and MBCs were between 10 and 640 μM. Ampicillin and erythromycin showed weak antibacterial activity with high MICs between 40 and 5,120 μM and MBCs between 160 and higher than 5,120 μM, indicating the presence of drug resistance. These results indicate that CATH-1,−3 and PMAP-36 have good bactericidal efficacy against *S. aureus, S. enteritidis*, and *E. coli*.

**Table 2:** Antibacterial activity of individual components of AMPs and antibiotics against *S. aureus, S. enteritidis*, and *E. coli*.

**MIC/MBC (μM) toward bacterial strains**						
	***S. aureus***		***S. enteritidis***		***E. coli***	
**Sample**	**MIC**	**MBC**	**MIC**	**MBC**	**MIC**	**MBC**
CATH-1	5	5	5	10	10	10
CATH-2	5	10	2.5	10	5	20
CATH-3	2.5	5	5	10	10	10
CATH-B1	>40	>40	>40	>40	>40	>40
CRAMP	20	>40	5	20	10	40
PMAP-36	10	10	5	5	5	5
PR-39	40	>40	2.5	10	10	10
Ampicillin	80	640	5,120	>5,120	160	160
Tetracycline	10	40	40	640	5	10
Gentamicin	20	40	2.5	5	1.25	2.5
Erythromycin	5,120	>5,120	80	>5,120	40	>5,120

### Antibacterial Effect of AMPs Combined With Antibiotics

**Figure 1:**
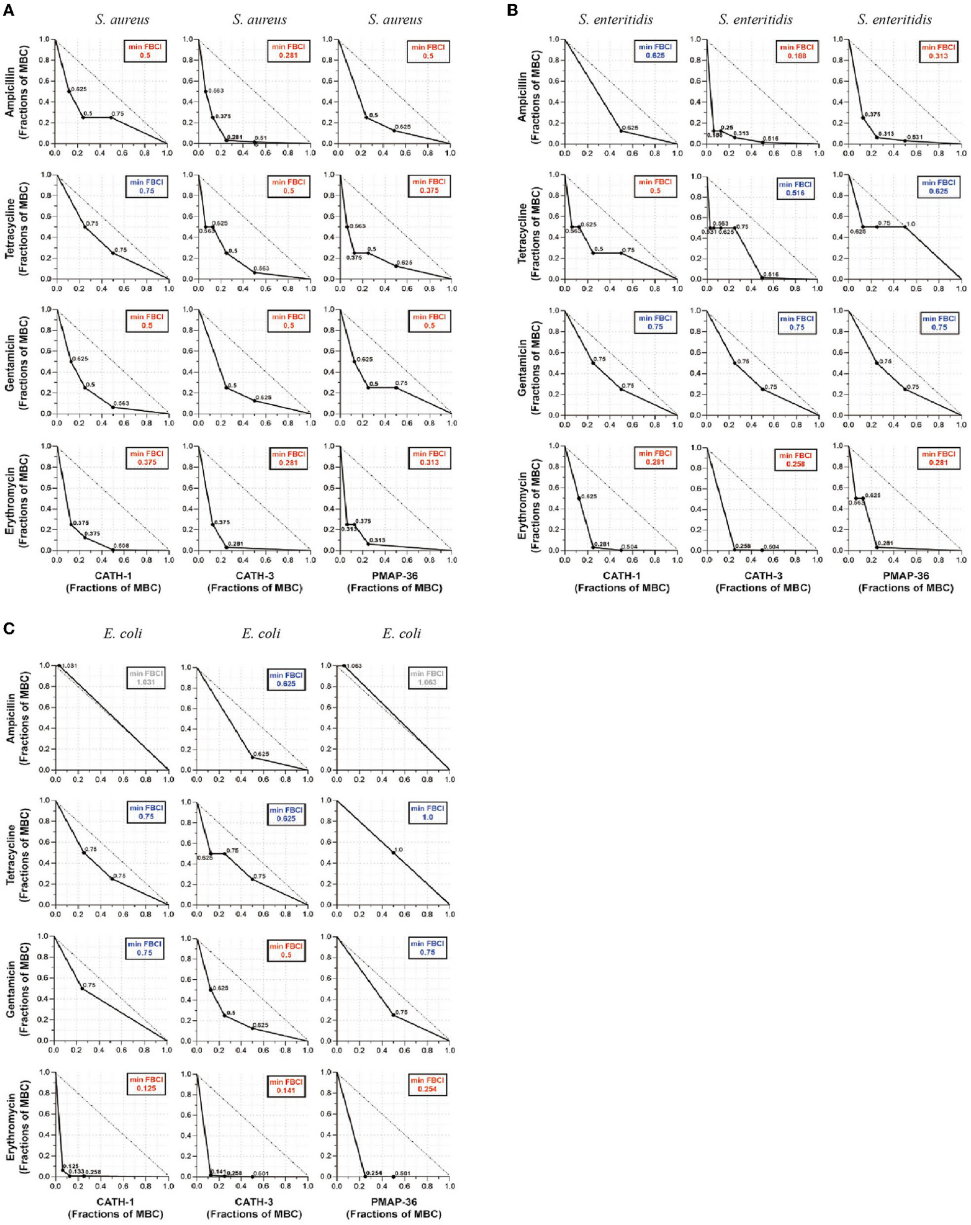
Antibacterial effect of antimicrobial peptides combined with antibiotics against *S. aureus, S. enteritidis*, and *E. coli*. The isotope map shows the antibacterial effect of the combination of AMPs (CATH-1, CATH-3, and PMAP-36) and antibiotics (ampicillin, tetracycline, gentamicin, and erythromycin) against *S. aureus* **(A)**, *S. enteritidis* **(B)**, and *E. coli* **(C)**. The synergic antibacterial activity was determined by the fractional bactericidal concentration index (FBCI). FBCI values were defined as synergy for FBCI < 0.5, additivity for 0.5 < FBCI ≤ 1, indifference for 1 < FBCI ≤ 2, and antagonism for FBCI > 2. In each contour map, the colors, including red, blue, and gray, represent synergy, additivity, and independence, respectively.

According to the MIC and MBC of AMPs, antibacterial effects of CATH-1,−3 and PMAP-36 combined with ampicillin, tetracycline, gentamicin, and erythromycin were determined using the broth microdilution checkerboard assay against *S. aureus, S. enteritidis*, and *E. coli*. FBCI was used to evaluate the antibacterial efficacy of AMPs and antibiotics. The results showed that FBCIs of only CATH-1 + tetracycline were higher than 0.5 against *S. aureus* (Figure 1A) while FBCIs of other combinations (CATH-1,−3 and PMAP-36 with ampicillin, tetracycline, gentamicin, and erythromycin) were lower 0.5. Furthermore, FBCIs of CATH-1,−3 and PMAP-36 combination with erythromycin were lower 0.5 against *S. enteritidis* and *E. coli* (Figures 1B,C). However, FBCIs of other combinations (CATH-1,−3 and PMAP-36 with ampicillin, tetracycline, and gentamicin) were higher than 0.5 or even higher than 1.0 against *S. enteritidis* and *E. coli*. These results demonstrate that the combination of CATH-1,−3 and PMAP-36 with erythromycin, respectively, has synergically antibacterial activity against *S. aureus, S. enteritidis*, and *E. coli*. Moreover, no antagonistic interaction was found.

### Time-Killing Kinetics of AMP Combination With Erythromycin

**Figure 2:**
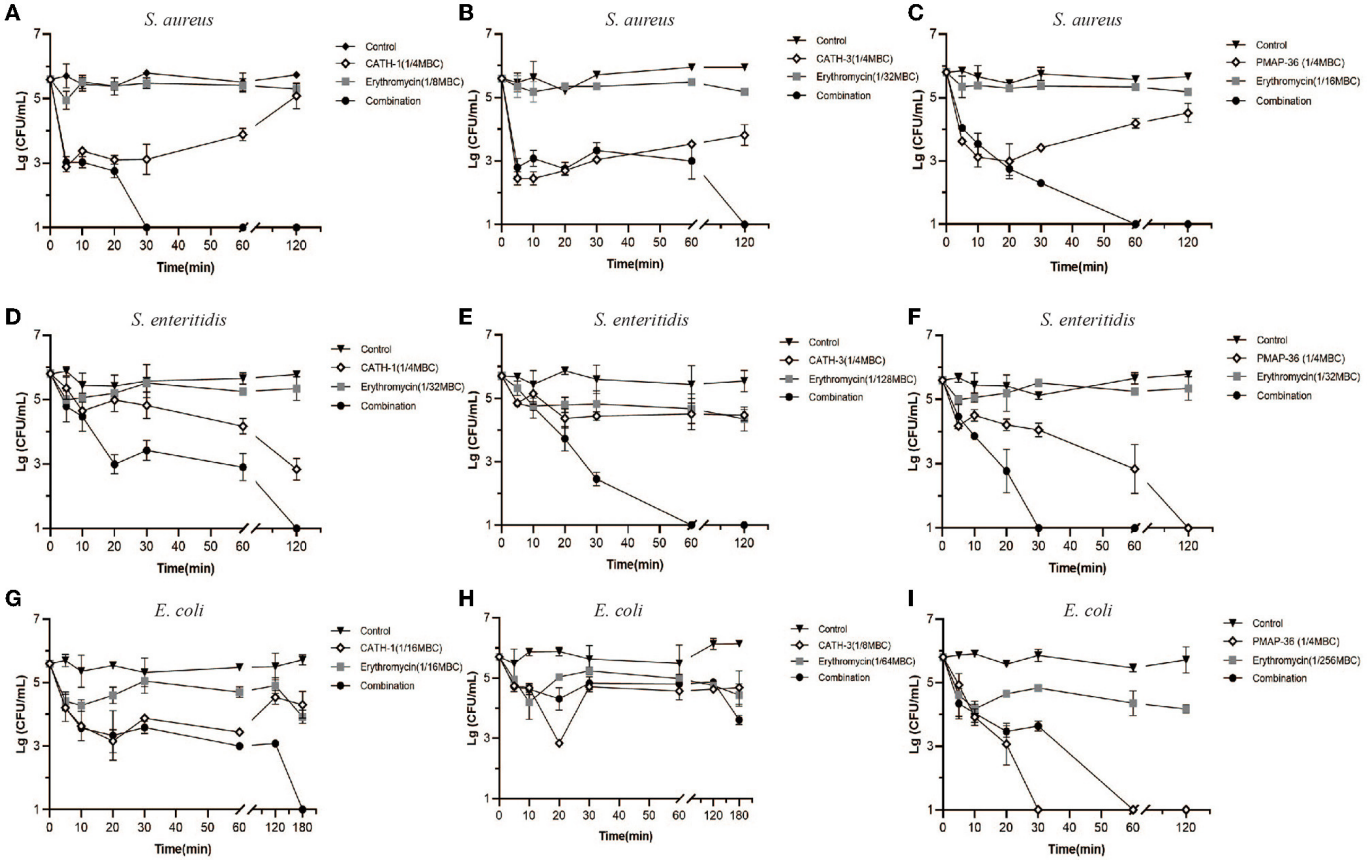
Time-killing kinetics of antimicrobial peptides combination with erythromycin. Time-killing kinetics of CATH-1, CATH-3, and PMAP-36 combination with erythromycin against *S. aureus* **(A–C)**, *S. enteritidis* **(D–F)**, and *E. coli* **(G–I)** are shown.

Time-killing assay was performed to examine the bactericidal abilities of CATH-1,−3 and PMAP-36 in combination with erythromycin against *S. aureus, S. enteritidis*, and *E. coli*. As shown in Figures 2A–I, AMPs (CATH-1 and CATH-3) and erythromycin alone at sub-MBC failed to show obvious bactericidal activity within 180 min against *S. aureus, S. enteritidis*, and *E. coli*, but AMPs in combination with erythromy cin at sub-MBC killed all the bacteria within 180 min, except for combination treatment of CATH-3 and erythromycin against *E. coli* (Figure 2H). CATH-1 in combination with erythromycin at sub-MBC killed *S. aureus, S. enteritidis*, and *E. coli* within 30, 120, and 180 min, respectively (Figures 2A,D,G). Notably, PMAP-36 alone at sub-MBC killed *S. enteritidis* and *E. coli* within 120 min and 30 min, respectively (Figures 2F,I), which is in contrast to the results that show PMAP-36's MBC (Table 2), indicating that PMAP-36 shows transient bactericidal activity at 120 and 30 min. These results indicate that CATH-1 combination with erythromycin at sub-MBC has sustained bactericidal activity against all three bacterial strains.

### Hemolytic Activity and Cytotoxicity

**Figure 3:**
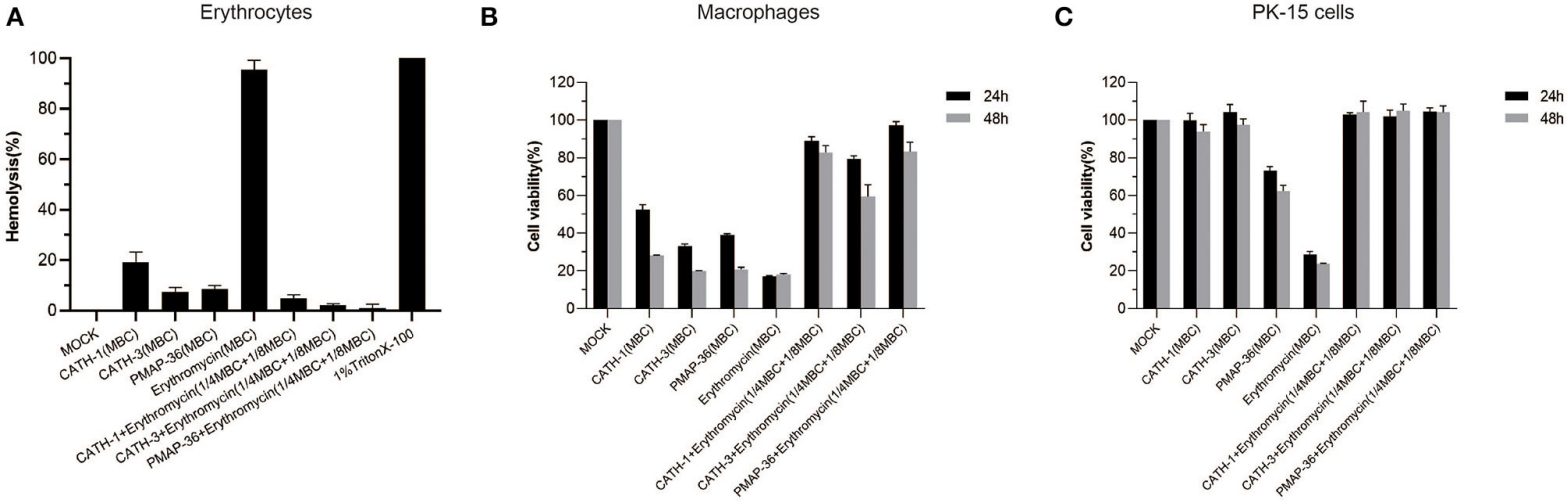
Hemolytic activity and cytotoxicity of antimicrobial peptides combination with erythromycin. Hemolytic activity of CATH-1, CATH-3, and PMAP-36 combination with erythromycin to the red blood cells of mice **(A)**. Cell viability of peritoneal macrophages **(B)** and PK-15 cells **(C)** treated with CATH-1, CATH-3, and PMAP-36 combination with erythromycin according to WST-1 assay.

To evaluate the toxicity of AMPs in combination with erythromycin, mice erythrocytes and macrophages and PK-15 cells were used to determine hemolytic activity and cytotoxicity, respectively. The results showed that CATH-1,−3 and PMAP-36 alone at MBC showed low hemolysis (<20%), and erythromycin alone at MBC showed high hemolysis (>95%) (Figure 3A). However, AMPs (CATH-1,−3 and PMAP-36) combined with erythromycin at sub-MBC showed lower hemolytic activity (<3%) (Figure 3A). Cytotoxicity assay results showed that CATH-1,−3, PMAP-36, and erythromycin alone at MBC showed toxicity to macrophages (cell viability <30%), while CATH-1 and CATH-3 alone at MBC did not show toxicity to PK-15 cells. PMAP-36 and erythromycin alone at MBC showed to some extent toxicity toward murine macrophages with 60–70% cell viability and PK-15 cells with less than 30% cell viability. However, AMPs (CATH-1,−3 and PMAP-36) combined with erythromycin at sub-MBC did not have toxicity to cells (Figures 3B,C). These results indicate that AMPs combination with erythromycin reduced hemolytic activity and cytotoxicity *via* the use of lower concentrations of erythromycin and AMPs.

### The Effect of CATH-1 and Erythromycin Combination on Bacterial Morphology

**Figure 4:**
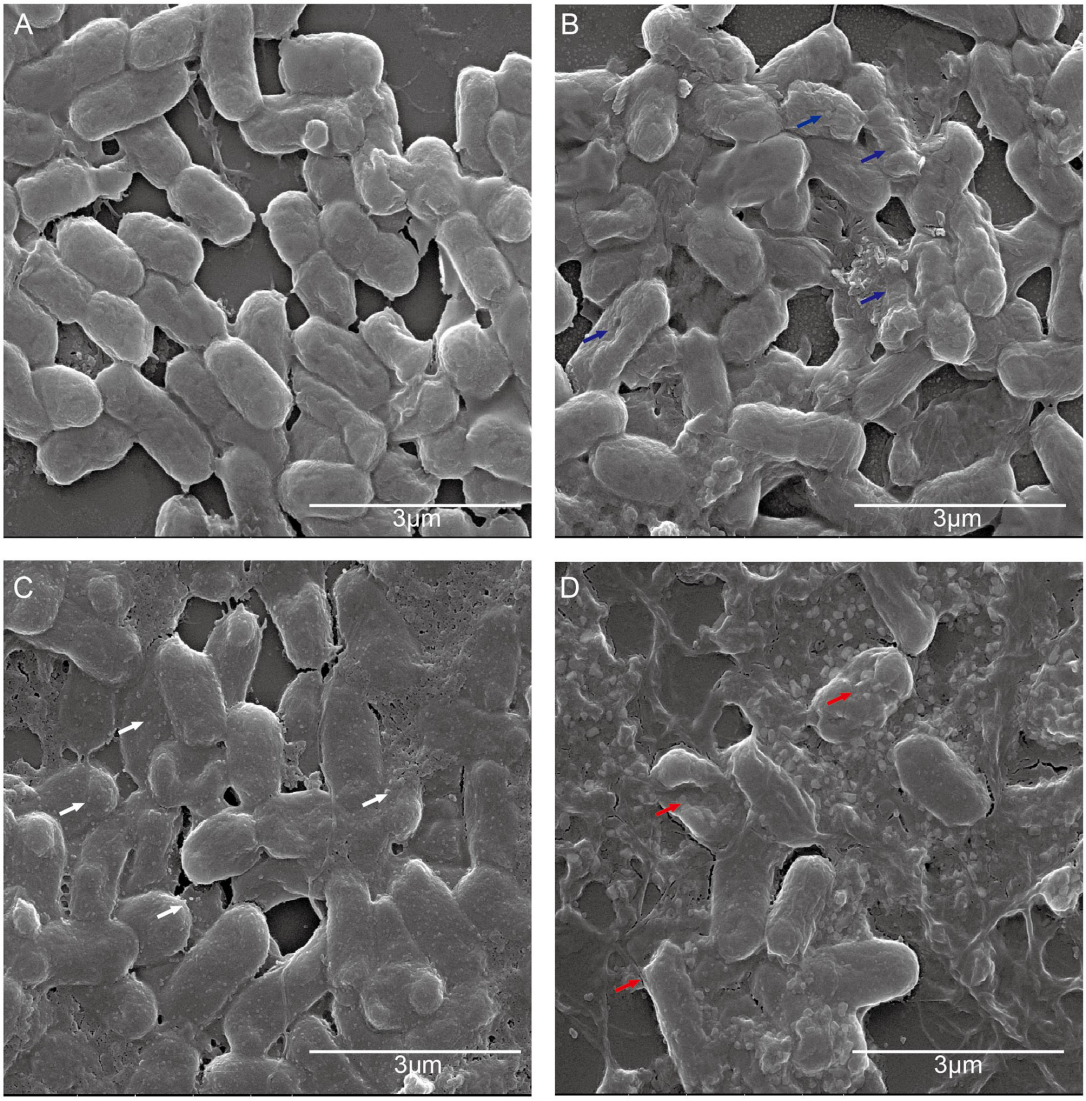
The effect of CATH-1 and erythromycin combination on *E. coli* morphology. *E. coli* cells were grown to the mid-logarithmic phase and then treated with CATH-1 and erythromycin. After treatment, SEM was performed to observe bacterial morphology. **(A)** Untreated *E. coli*. **(B)** 1/16 MBC CATH-1-treated *E. coli*. **(C)** 1/16 MBC erythromycin-treated *E. coli*. **(D)** 1/16 MBC CATH-1 + 1/16 MBC erythromycin-treated *E. coli*. Blue arrows represent rough cell membrane, white arrows represent micelles, and red arrows represent cell lysis.

To further investigate the antibacterial mechanism of CATH-1 and erythromycin combination, *E. coli* cellular morphology was observed by SEM after CATH-1 and erythromycin treatment. The results showed that untreated cells showed intact membrane (Figure 4A) and the membrane of CATH-1-treated cells became rough (Figure 4B, blue arrows). Erythromycin induced the formation of micelles (Figure 4C, white arrows). In addition, the combination treatment of CATH-1 and erythromycin caused bacterial cell lysis (Figure 4D, red arrows). These results indicate that CATH-1 and erythromycin combination at sub-MBC enhances bactericidal activity by promoting cell damage.

### The Effect of CATH-1 and Erythromycin Combination on Bacterial Motility

**Figure 5:**
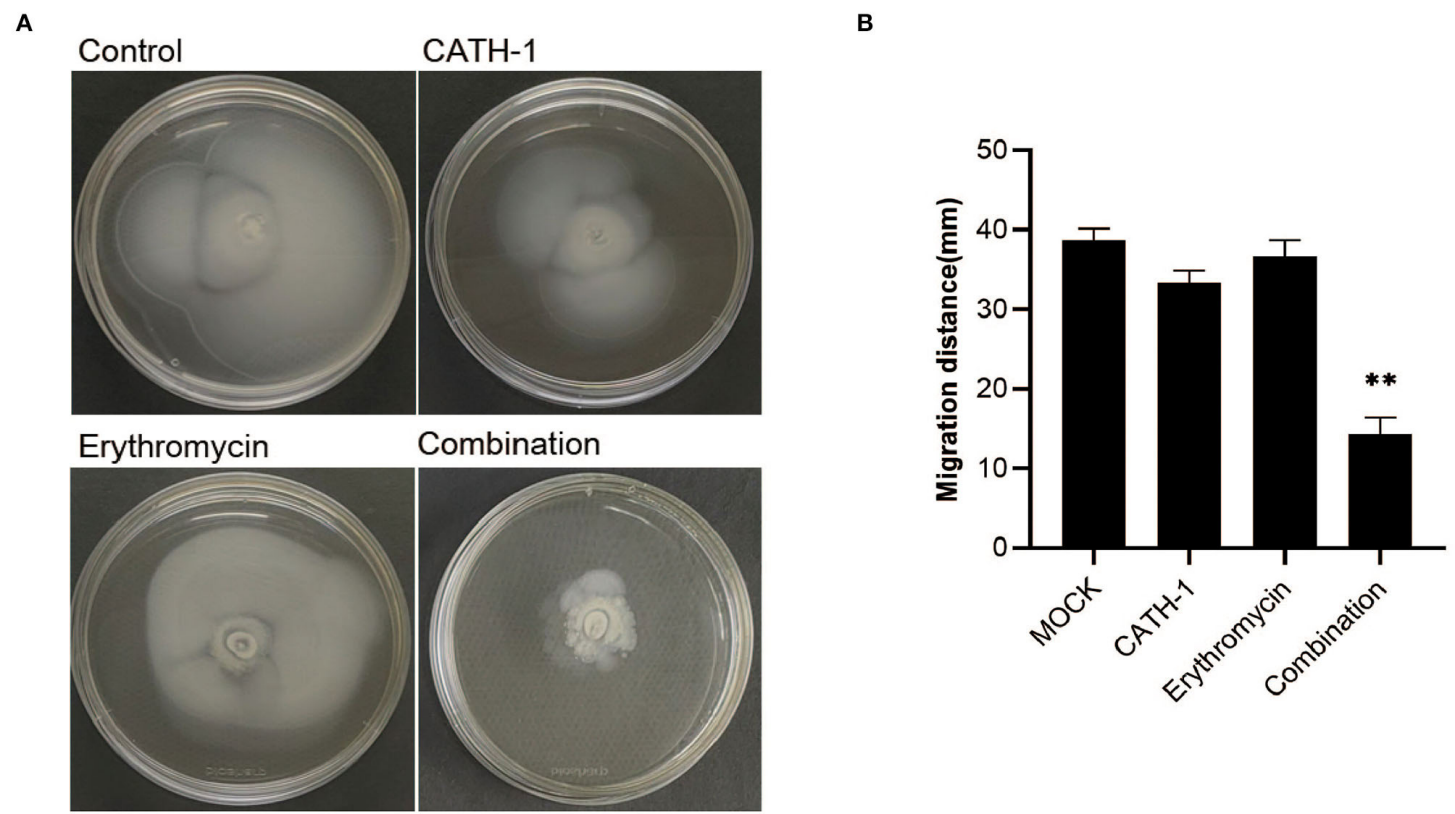
The effect of CATH-1 and erythromycin combination on bacterial motility. Mid-logarithmic phase *E. coli* were treated with CATH-1, erythromycin, and CATH-1 + erythromycin, and then *E. coli* was inoculated in the center of the semisolid agar. The inoculated plates were incubated at 37°C for 18 h. The motility image is shown **(A)**. The migration distance (mm) was measured **(B)**. Asterisks (*) indicate significant difference (* *p* < 0.05, ** *p* < 0.01).

Next, the effect of CATH-1 and erythromycin on the motility of *E. coli* was investigated using semisolid agar. As shown in Figure 5, after 18 h incubation, the bacterial migration distance was significantly reduced in semisolid agar plates containing CATH-1 in combination with erythromycin at sub-MIC (Figure 5A) while CATH-1 and erythromycin alone did not affect bacterial migration distance. Specifically, the inhibited motor area of *E. coli* treated with CATH-1 in combination with erythromycin was 14.33 mm, which was 62.94% smaller than that of the control (Figure 5B). These results indicate that the CATH-1 and erythromycin combination significantly reduced bacterial motility, thereby promoting bactericidal activity.

### The Effect of CATH-1 and Erythromycin Combination on Bacterial Drug Resistance

**Figure 6:**
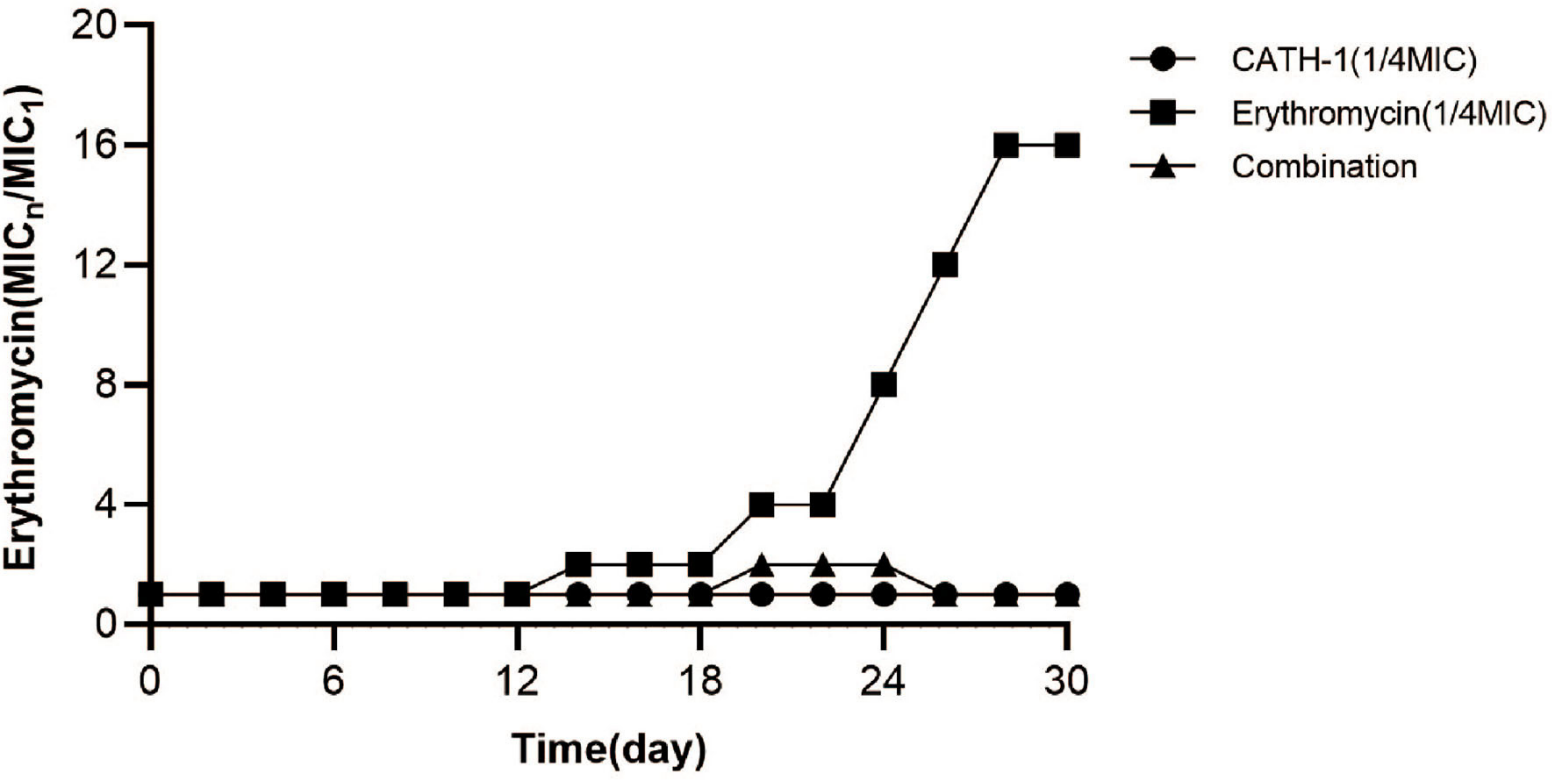
The effect of CATH-1 and erythromycin combination on bacterial drug resistance. The *E. coli* strain grown independently with CATH-1 (1/4 MIC), erythromycin (1/4 MIC), and CATH-1 (1/8 MIC) combination with erythromycin (1/16 MIC) at 37°C for consecutive 30 generations. A fixed concentration of CATH-1 (equivalent to 1/4 MIC) was added to the 2-fold increasing concentration of erythromycin. Bacteria from the highest concentration of drug combination were regrown, and MIC of erythromycin was measured, and then treated them with the drug combination again. The change in MIC was described by normalizing the MIC of *n* generation to the MIC of first generation.

The generation of bacterial drug resistance is often related to the improper use of antimicrobials. To explore this problem, *E. coli* were exposed to a sub-MIC concentration of CATH-1 and erythromycin for 30 passages. As shown in Figure 6, the drug resistance of *E. coli* in the sub-MIC solution of erythromycin appeared in the 14th generation, and MIC increased by 2-fold. After 30 generations, the MIC of erythromycin increased by 16-fold, indicating the induction of the resistant gene to erythromycin. On the contrary, *E. coli* cultured in the sub-MIC solution of CATH-1 did not show drug resistance after 30 passages. In the case of coexistence of CATH-1 and erythromycin, the MIC increased by only 2-fold in the 20th generation, and the MIC after 30 generations was 16-fold lower than that of erythromycin alone, indicating that additive CATH-1 can delay the production of erythromycin resistance.

## Discussion

Multidrug-resistant pathogens have been a great challenge for human and animal health, and a steady decline in the discovery of new antibiotics makes it difficult to develop new therapies to control infection (Nørgaard et al., 2019). Therefore, there is an urgent need for alternative strategies to kill pathogens without the production of drug resistance. One promising strategy is the use of AMPs with direct microbicidal properties (Li et al., 2016). It has been reported that AMPs from chicken (CATH-1,−2,−3, and -B1), porcine (PMAP-36 and PR-39), and mice (CRAMP) have shown a broad spectrum of antimicrobial activities (Chromek et al., 2012; Veldhuizen et al., 2013, 2014). In this study, we found that CATH-1, CATH-3, and PMAP-36 had a synergistic effect with erythromycin on bacterial killing and significantly reduced resistance to antibiotics.

It has been reported that the bactericidal mechanism of AMPs acts through the cell membrane not involved in a specific target, so it is unlikely to develop microbial resistance (Drayton et al., 2021). Our study showed that AMPs (CATH-1,−3 and PMAP-36) have good bactericidal activities against *S. aureus, S. enteritidis*, and *E. coli*, which is consistent with other findings that CATH-1,−3 and PMAP-36 showed a broad spectrum of bactericidal activities against *S. aureus, S. enteritidis, E. coli, Campylobacter jejuni*, and *Clostridium perfringens* (Lv et al., 2014; Nguyen et al., 2021). Importantly, Veldhuizen and Brouwer et al. found that CATH-1 and CATH-3 did not induce bacterial resistance against *S. aureus* and *Klebsiella pneumoniae* (Veldhuizen et al., 2013). It has been found that PMAP-36's analogs exhibited an impressive therapeutic effect *in vivo* against *Salmonella choleraesuis* and *Listeria monocytogenes* (Zhou et al., 2019). These results demonstrate that it is promising to develop AMPs as alternatives or adjuvants to antibiotics.

Antimicrobial peptides combined with conventional antibiotics have been the proposed strategy to reduce bacterial resistance. The combination of peptide Ud and rifampin reduced the MBC of peptides and antibiotics against *E. coli* (Anantharaman et al., 2010). Our study showed that the combination of CATH-1 and erythromycin reduced the MBC of the peptide by at least 4-fold against *S. aureus, S. enteritidis*, and *E. coli*, as well as even 16-fold MBC reduction of erythromycin against *E. coli*. Importantly, the combination of PMAP-36 and erythromycin even induced a 256-fold reduction in the MBC of erythromycin against *E. coli*. These combinations of AMPs and erythromycin exhibited high bactericidal abilities within 3 h against bacteria, although combination of CATH-3 and erythromycin failed to kill *E. coli* within 3 h. These results indicate that the combination of CATH-1 and PMAP-36 with erythromycin provides an attractive option for the treatment of bacterial infections.

Most AMPs exhibit bactericidal activity through the disruption of cell membranes, resulting in cytoplasmic leakage and cell death (Teixeira et al., 2012). Zhao et al. showed that plectasin-derived peptide MP1102 killed *Streptococcus suis* serotype 2 by destroying the cell membrane integrity, resulting in the penetration of MP1102 into bacterial cells and interaction with cell DNA (Zhao et al., 2019). CATH-2 has been reported to kill *S. aureus* and *E. coli* by penetrating into the cell membrane, resulting in the disruption of the synthesis of DNA and protein (Schneider et al., 2016, 2017). Similarly, our study showed that CATH-1 from the same species as CATH-2 showed slight damage in bacterial cell membrane under 1/16 MBC but combination with erythromycin completely induced bacterial cell fragmentation. In addition, the combination of CATH-1 and erythromycin significantly blocked bacterial motility. Previous studies have shown that AMPs disrupted cell membranes and contributed to the penetration of antibiotics and then interaction with an intracellular target such as DNA and protein, which improved the bactericidal efficacy (Gupta et al., 2015; Wu et al., 2017; Zharkova et al., 2019). Together, we reasonably speculated that CATH-1 disrupts the bacterial cell membrane and promotes the uptake of erythromycin, which inhibits bacterial protein synthesis and finally induces cell death.

Nowadays, new antimicrobial compounds are required to address the challenge of bacterial multidrug resistance. It has been reported that methicillin, tigecycline, colistin, and vancomycin induced drug resistance genes in *S. aureus* and *E. coli* (Foster, 2017; Zhou et al., 2020). However, AMPs are difficult to induce drug resistance (Veldhuizen et al., 2013; Manniello et al., 2021). It has been reported that AMP M(LLKK)_2_M combined with rifampicin can reduce the drug resistance of mycobacteria to rifampicin (Khara et al., 2014), which is similar to our findings that CATH-1 combined with erythromycin can reduce the drug resistance of *E. coli* to erythromycin. These results indicate that the combination of AMPs with antibiotics can delay bacterial drug resistance and provides an attractive option for preventing the induction of bacterial drug resistance.

It can be concluded that the combination of CATH-1 and PMAP-36 with erythromycin reduced the MBC of each antiagents and exhibited high bactericidal activity against *S. aureus, S. enteritidis*, and *E. coli*. Furthermore, CATH-1 combination with erythromycin completely induced bacterial cell fragmentation and reduced the production of bacterial drug resistance. Our study provides valuable information about the combination of AMPs and antibiotics, which will contribute to the development of antibiotics adjuvant.

## Data Availability Statement

The original contributions presented in the study are included in the article/supplementary material, further inquiries can be directed to the corresponding authors.

## Ethics Statement

The animal study was reviewed and approved by Institutional Animal Care and Use Committee (IACUC) of Southwest University, Chongqing, China.

## Author Contributions

YL, HT, RC, QL, and KJ performed experiments. CY, HC, and D-LH helped analyze the data. RF and LP supervised the study and designed the experiments. YL, LP, D-LH, and RF drafted the manuscript. All authors have read and agreed to the published version of the manuscript.

## Funding

This study was supported by the National Natural Science Foundation of China (32102684, 32172850), the Fundamental Research Funds for the Central Universities (SWU120057), the Chongqing Science and Technology Commission (cstc2021jcyj-msxm2218, cstc2021jcyj-msxmX0504), the Chongqing Pig Industry Technology System (20211105), and the Foundation for Innovation Research Groups in Chongqing Universities (CXQT20004).

## Conflict of Interest

The authors declare that the research was conducted in the absence of any commercial or financial relationships that could be construed as a potential conflict of interest.

## Publisher's Note

All claims expressed in this article are solely those of the authors and do not necessarily represent those of their affiliated organizations, or those of the publisher, the editors and the reviewers. Any product that may be evaluated in this article, or claim that may be made by its manufacturer, is not guaranteed or endorsed by the publisher.
